# Indigenous medicinal plants used in folk medicine for malaria treatment in Kwara State, Nigeria: an ethnobotanical study

**DOI:** 10.1186/s12906-023-04131-4

**Published:** 2023-09-16

**Authors:** Ikponmwosa Owen Evbuomwan, Oluyomi Stephen Adeyemi, Olarewaju Michael Oluba

**Affiliations:** 1 SDG #03 Group – Good Health and Well-being, Landmark University, Ipetu Road, PMB 1001, Omu-Aran, 251101 Nigeria; 2 Department of Biochemistry, Landmark University, Ipetu Road, PMB 1001, Omu-Aran, 251101 Nigeria; 3 Department of Microbiology, Cellular Parasitology Unit, Landmark University, Ipetu Road, PMB 1001, Omu-Aran, 251101 Nigeria; 4 Laboratory of Sustainable Animal Environment, Graduate School of Agricultural Science, Tohoku University, 232-3 Yomogida, Naruko-Onsen, Osaki, Miyagi 989-6711 Japan

**Keywords:** Traditional medicine, Traditional medicine practitioners, *Mangifera indica*, Decoction, Conservation status, Antimalarial compounds

## Abstract

**Background:**

Folk medicine is crucial to healthcare delivery in the underdeveloped countries. It is frequently used as a primary treatment option or as a complementary therapy for malaria. Malaria is a deadly disease which greatly threatens global public health, claiming incredible number of lives yearly. The study was aimed at documenting the medicinal plants used for malaria treatment in folk medicine in Kwara State, Nigeria.

**Methods:**

Ethnobotanical information was collected from selected consenting registered traditional medicine practitioners (TMPs) through oral face-to-face interviews using in-depth, semi-structured interview guide. The ethnobotanical data were analysed, and descriptive statistical methods were used to compile them.

**Results:**

Sixty-two indigenous medicinal plants, including 13 new plants, used for malaria treatment were identified in this study. The TMPs preferred decoction in aqueous solvent (34%) and steeping in decaffeinated soft drink (19%) for herbal preparations. Oral administration (74%) was the main route of administration, while leaves (40%) and stem barks (32%) were the most dominant plant parts used in herbal preparations. The most cited families were Fabaceae (15%) and Rutaceae (6%), while *Mangifera indica* (77.14%), *Enantia chlorantha* (65.71%), *Alstonia boonei* (57.14%) followed by *Cymbopogon citratus* (54.29%) were the most used plants. Besides, the antimalarial activities of many of the plants recorded and their isolated phytocompounds have been demonstrated. Furthermore, the conservation status of 4 identified plants were Vulnerable.

**Conclusion:**

The study showed strong ethnobotanical knowledge shared by the TMPs in the State and provides preliminary information that could be explored for the discovery of more potent antimalarial compounds.

**Supplementary Information:**

The online version contains supplementary material available at 10.1186/s12906-023-04131-4.

## Background

Malaria is a deadly disease which has continued to plague global health for many centuries now, leading to an unimaginable loss of life annually. The malaria scourge is evolving, dynamic and diverse, and currently, it is concentrated in some of the poorest nations in the world [1]. In particular, the World Health Organization (WHO) African Region is affected the most where it causes huge economic setbacks, and mostly afflicts children below age 5; an outcome that is largely attributed primarily to *Plasmodium falciparum*, the most virulent of the five human malaria parasite species [2–4]. In 2021, an estimated 247 million malaria cases were reported in 84 malaria endemic countries worldwide and Africa, with 234 million cases, accounted for approximately 95% of all malaria cases globally. Death due to malaria in 2021 was estimated to be 619, 000 and the largest mortality was recorded in Nigeria [4]. The rapid spread of resistance of the malaria parasites to recommended drugs including artemisinin-based compounds [4–8], in addition to the high cost of antimalarial drugs and counterfeiting have made treatment and control of the disease very challenging and almost impossible [9]. Hence, this necessitates urgent need for more potent and safer alternative therapeutic agents with novel mode of action.

Since time immemorial, humans have relied upon medicinal plants for the prevention and cure of myriad of diseases and pathological disorders including malaria [10]. The application of the knowledge of medicinal plants for the treatment of various diseases has attracted the attention of researchers and formed the basis for modern pharmacology leading to the discovery and development of different therapeutic agents with plant origin [11, 12]. Today, the application of medicinal plants in folk medicine is still accepted as a preferred source of primary health care delivery in many nations including Nigeria and other parts of Africa [13–16] in spite of the paucity of pharmacological elucidation of their mechanisms of action and standard clinical trials. About 80% of the world’s population is reported to depend on the use of medicinal plants as essential sources of pharmaceutical and therapeutic needs largely because they are accessible and affordable [17–19].

Nigeria has a rich heritage in folk medicine in which herbal preparations comprising different medicinal plant parts are used as an alternative or to complement orthodox medicine in the prevention or treatment of many diseases and health disorders [18, 20, 21]. Till date, many Nigerians, especially those in rural and peri-urban areas, still depend on folk medicine as a major source of health care because it is readily available, conventional medicine is expensive, there is lack of adequate health care facilities, and also due to their limited access to allopathic medicine [13, 22]. Yet, others use herbal formulations most of the time simply because they trust them and their ancestors have been using them over the years.

A large number of medicinal plants that are used for malaria treatment in Nigeria, either singly or as polyherbal recipes, have been reported in previous studies [23–26]. Many of these plants have been demonstrated to possess antimalarial properties [27–30] and could serve as new leads for the discovery, design and development of more potent antimalarial agents [27]. This is predicated on the fact that the two most successful antimalarial drugs -quinine and artemisinin- were synthesised from plants [31, 32]. In the light of the above, exploration of the rich biodiversity of indigenous medicinal plant taxa through ethnobotanical and pharmacological studies becomes very important.

Ethnobotanical studies concentrate on the intricate relationship between indigenous people and local plants, including customs and cultural beliefs connected to various uses [33]. These studies help to collect vital ethnobotanical information from indigenous people including herbal practitioners in order to preserve the indigenous knowledge on the diagnosis of diseases, plant species used for treatment of diseases in folk medicine, their modes of preparation and administration, as well as the socio-cultural heritage of indigenous people for succeeding generations [34, 35]. In addition, ethnobotanical surveys are of great socio-economic importance to researchers as they are acknowledged as one of the most efficient ways of finding and documenting new medicinal plants with novel therapeutic properties and uses, and therefore enhance drug discovery and development approaches [36, 37]. This knowledge could be eroded and eventually lost to future generations without prompt and proper documentation since they are often passed through verbal communication and inappropriately documented [38, 39]. Besides, many of these plants are being destroyed due to industrialisation, urbanisation and expansion of housing programmes while some taxa are becoming endangered or threatened as a result of the loss of their natural habitats [40, 41].

The current study is premised on the observations of the historic application of medicinal plants in traditional medicine for the treatment of various human diseases among traditional medicine practitioners (TMPs) in the study area. We hypothesised that the TMPs that provide treatment for different human diseases and health disorders in the study area have valuable knowledge about the medicinal plants used for malaria treatment. Hence, this ethnobotanical survey was designed to identify and coherently document the indigenous medicinal plant taxa that are used in folk medicine for malaria treatment in Kwara State, Nigeria. Although, ethnobotanical studies of medicinal plants used in the treatment of malaria in north-eastern [42], south-western [25, 43–46], and south-eastern [23, 47, 48] regions of Nigeria have been reported, to the best of our knowledge, this is the first comprehensive account of the ethnobotanical resource of indigenous medicinal plants used for malaria treatment in the region, North Central Nigeria. The current survey was carried out as a preliminary to a larger study, the aim of which is to collect the five most used plants by the TMPs in the State for malaria treatment, evaluate them for antimalarial activity and isolate the bioactive principles in an effort to discovering new lead structures.

## Methods

### Description of the study area

**Fig. 1:**
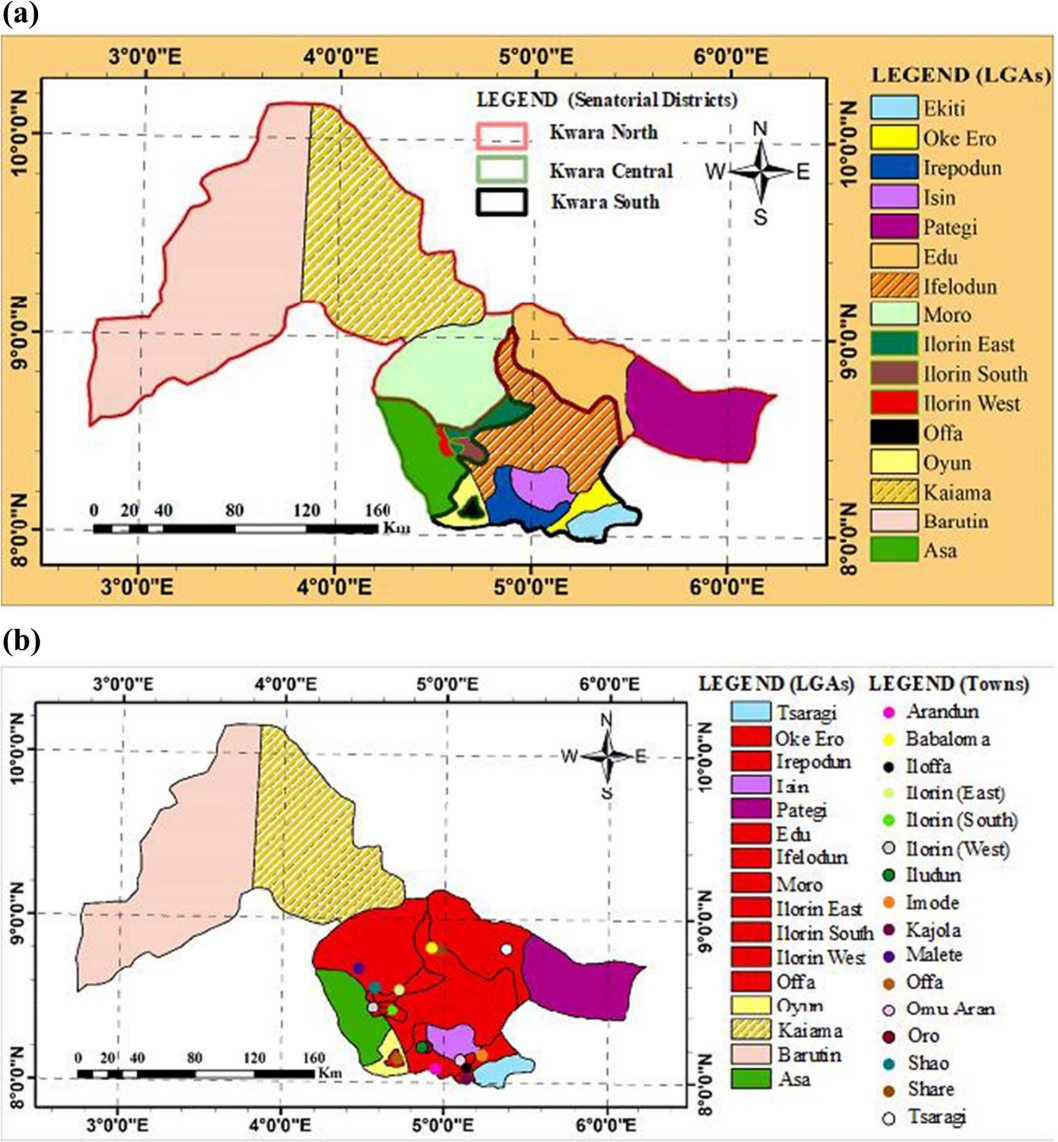
Map of Kwara State showing (**a**) the Senatorial Districts and LGAs, and (**b**) the LGAs (red) with the Towns/Villages visited

Kwara State is located in the North-Central region of the Federal Republic of Nigeria and lies between latitudes 7°45′ N and 9°30′ N and longitudes 2°30′ E and 6°25′ E. The state consists mostly of wooded savannah with forested regions in the south. It has a tropical wet and dry climate with a mean annual precipitation of about 1200 mm [49]. The distribution of rain is bimodal with long rains between April and September and short rains from October to November annually. The dry season is usually between November and March [50]. Its average annual temperature is about 26.2 ℃ and peaks at about 30 ℃ in March. Kwara State is divided into three Senatorial Districts: Kwara North, Kwara Central and Kwara South, and 16 Local Government Areas (LGAs) (Fig. 1).

The inhabitants of the State are predominantly of Yoruba-speaking ethnic group comprising Christians, Muslims and African Traditional worshippers. Others include Nupe, Bariba, Busa and Fulani minorities. They are dominated with farmers, traders, wage earners as well as those engaged in commercial services.

### Selection of study participants and exclusion criteria

The respondents (Traditional medicine practitioners, TMPs) were purposively selected from the study area adopting the method of Vasileiou et al. [51]. In each location, the highest-ranking TMP was located with the help of the indigenous field assistants. We explained the aim of the study to the highest-ranking TMPs and got a verbal approval from them for the study. They subsequently gave us the list and contacts of other TMPs in the communities. All the TMPs willing to participate in the study were included in the survey. They were questioned individually on their knowledge of malaria and indigenous medicinal plant species used for malaria treatment.

Hawkers of medicinal plants used for malaria treatment were excluded from the survey. Also, indigenes and dwellers with some form of knowledge about medicinal plants used for malaria treatment were excluded from the study.

### Ethnobotanical data collection and plant identification

The participants were questioned individually in their homes and workplaces on the knowledge of using medicinal plants for malaria treatment. Data on socio-demographic characteristic of the TMPs, diagnosis/symptoms of malaria and ethnobotanical uses of the indigenous medicinal plants were collected through oral face-to-face interviews by the use of in-depth, standardised semi-structured interview guide (Supplementary 1) [52]. The instrument was developed with the help of a statistician and sociologists in the Departments of Physical Sciences, and Sociology, respectively, Landmark University, Omu-Aran, Nigeria. However, the instrument was not pre-tested before its deployment since we used purposive sampling technique and did not anticipate any difficult problems during the survey. Also, the questions were easy to understand and not complex.

The survey was conducted between November and December, 2021. Ethnobotanical information including the vernacular names of commonly used plants for malaria treatment, frequently used plant parts, methods of herbal preparation, mode of administration, dosage, duration of malaria treatment and possible side effects were obtained from the participants and recorded using a recorder. They were later transcribed verbatim by the field assistants and properly documented. The bio-data of the participants including age, gender, town or village, religious affiliation, level of education and years of experience were also recorded. Three Yoruba-speaking field assistants, who are familiar with some of the medicinal plants used by the indigenous people of the State for malaria treatment, were engaged to administer and interpret the questions to the participants in their local languages, including Yoruba and Nupe, so as to facilitate efficient communication.

Photographs of the plants reported were taken to verify the taxonomic identification. Several plant identification guides were used to identify the family and species names of the medicinal plants obtained from the informants [53–55]. The scientific names of the plant species were checked with the “Plant List database” (www.theplantlist.org) [56] for accuracy. Thereafter, the voucher specimens were prepared and authenticated by experts, and deposited at the Forestry Research Institute of Nigeria (FRIN), Ibadan, National Institute of Pharmaceutical Research and Development (NIPRD), Abuja and University of Benin Herbarium (UBH), Benin City, Nigeria.

All methods were carried out in accordance with the relevant guidelines and regulations as stipulated in the International Union for Conservation of Nature (IUCN) Policy Statement on Research Involving Species at Risk of Extinction as well as the Convention on International Trade in Endangered Species (CITES) of Wild Fauna and Flora.

### Data analysis

Data were computed and analysed using the Statistical Packages for the Social Sciences (IBM SPSS) Statistics software v25. Descriptive statistical method (percentage and/or frequency) was employed to summarise the ethnobotanical data. Results were reported as charts and tables.

### Conservation status of the medicinal plants

The conservation status of the indigenous medicinal plants was collected and recorded for different conservation attributes, and classified according to the IUCN [57].

## Results and discussion

### Sociodemographic data of the informants

For the survey interview, a total of 35 indigenous TMPs comprising 10 (28.57%) males and 25 (71.43%) females (Supplementary 2) were randomly selected from 16 Towns/Villages across 9 Local Government Areas (LGAs) in the 3 Senatorial Districts of Kwara State. The educational background of the informants showed that 17 (48.6%), had no formal education. Seven of the informants had secondary (20%) and 6 had primary education (17.1%) respectively. Others attended Polytechnic (8.6%) and College of Education (5.7%).

The higher number of female respondents involved in herbalism could be attributed to the unique role women play both in the family and society. A similar trend was also observed in earlier reports [45, 58, 59]. The largest age group of the informants was 48–69 years old (51.43%); this was followed by those above or equal to 70 years old (25.71%) and 25–47 years old (20%) while younger TMPs less than 21 years old comprised the lowest percentage (2.86%) of informants. This corroborates earlier studies by Tchicaillat-Landou et al. [59] and Raimi et al. [60]. They reported that the majority of traditional healers who served as respondents in their ethnobotanical study was 40 and above. Also, our findings substantiate the report of Mudau et al. [61] who showed that most of the traditional healers in their survey were 41 years and above with adults between 21 and 40 years constituting the least.

Put together, the data support the concept that older generations are the main custodians of the knowledge of folk medicine and are more interested in traditional medicine practice compared with the younger generations. This development presents an enormous threat to the sustainable retention, transfer and enhancement of existing knowledge about the use of indigenous plants for folk medicine since it may eventually be lost following the demise of older generation. A possible reason for lower knowledge among young informants could be as a result of their limited interest in herbalism due to changes in lifestyle that are influenced by rapid sociocultural transformation, marked by industrialisation and modernisation. Also, due to the increasing literacy level, traditional medicine practice is becoming unpopular and unattractive to younger generations as such, they do not pay attention to the knowledge of using medicinal plants as sources of therapy.

Eleven (31.43%) of the informants had over 40 years’ experience as TMPs. This is followed by 13 (37.14%), 7 (11.25%) and 4 (11.43%), for 26–40, 11–25, and less than 10 years of experience, respectively. Regarding religious affiliation, most of the informants were Muslims (77%), followed by Christians (17%) and Traditionalist (6%). When we asked our informants how they gathered knowledge about plants and became TMPs, 48.6% stated that they were taught folk use of medicinal plants by their mother. Others received their education about herbal medicine from their father (25.7%), maternal grandparents (17.1%), maternal aunts (5.7%), and 2.9% was self-taught. This shows that transition of knowledge about the use of medicinal plants as sources of therapy is usually ancestral; passed down from one generation to another, supporting earlier findings [62, 63].

### Documented medicinal plant species used for malaria treatment in Kwara State and their taxonomy

The ethnobotanical information inventoried in this survey is presented in Table 1. The vernacular/indigenous, common and scientific names of the plants, plant family, plant habit, plant parts used, use reports and citation frequency (%) are shown in the table. In this ethnobotanical investigation, a total of 62 indigenous medicinal plant species with their voucher numbers (Supplementary 3), belonging to 58 genera distributed across 36 families were revealed by the TMPs to be used in traditional health care system for malaria treatment (Table 1).

**Table 1:** Ethnobotanical data including use reports, citation frequency and conservation status of the identified indigenous medicinal plants

S/n	Local names	Common names	Scientific names Voucher number	Plant family	Habit of plant	Plant parts used	Use value	Citation frequency (%)	Conservation status
1.	Mongoro	Common Indian mango	*Mangifera indica* Linn (FHI 109451)	Anacardiaceae	Tree	Stem bark, root, leaves	27	77.14	DD
2.	Awopa or Dokita igbo	African yellow wood	*Enantia chlorantha* Oliv (FHI 109950)	Annonaceae	Tree	Stem bark, root, leaves	23	65.71	NE
3.	Ahun	Stool wood, Pattern wood	*Alstonia boonei* De Wild (FHI 107254)	Apocynaceae	Tree	Stem bark, root	20	57.14	LC
4.	Ewe tii	Lemon grass	*Cymbopogon citratus* (DC.) Stapf (FHI 605214)	Poaceae	Grass	Leaves	19	54.29	NE
5.	Egbesi	African peach	*Nauclea latifolia* Sm (FHI 112779)	Rubiaceae	Tree	Stem bark, root, leaves	15	42.86	LC
6.	Oruwo	Brimstone tree	*Morinda lucida* Benth (FHI 106992)	Rubiaceae	Tree	Stem bark, root, leaves	10	28.57	LC
7.	Ibepe	Pawpaw	*Carica papaya* Linn (FHI 109462)	Caricaceae	Tree	Leaves, Unripe fruit	9	25.71	DD
8.	Kasu	Cashew	*Anacardium occidentale* Linn (FHI 109858)	Anacardiaceae	Tree	Stem bark, root, leaves	9	25.71	LC
9.	Aganwo or Oganwo	African mahogany	*Khaya ivorensis* A. Chev (FHI 56845)	Meliaceae	Tree	Stem bark	9	25.71	VU
10.	Boonii	Gum Arabic tree	*Acacia nilotica* (L.) Delile (FHI 108425)	Fabaceae	Tree	Stem bark, leaves	8	22.86	LC
11.	Amuje wewe or Kanti-kanti	Crimson thyme	*Byrsocarpus coccineus* Schum. & Thonn (FHI 112950)	Connaraceae	Shrub	Stem bark	6	17.14	LC
12.	Epoara	Sleeping morning	*Waltheria indica* Linn (FHI 92465)	Malvaceae	Shrub	Stem bark, leaves	6	17.14	NE
13.	Koko	Cocoa	*Theobroma cacao* Linn (FHI 700314)	Sterculiaceae	Tree	Stem bark	6	17.14	NE
14.	Laali (Yoruba), Lǎli (Nupe)	Henna tree	*Lawsonia inermis* Linn (FHI 702616)	Lythraceae	Shrub	Leaves	5	14.29	LC
15.	Akerejupon or Ajo	Sphenocentrum	*Sphenocentrum jollyanum* Pierre (FHI 108283)	Menispermaceae	Shrub	Root, leaves	5	14.29	NE
16.	Osan wewe	Lime	*Citrus aurantifolia* (Christm.) Swingle (FHI 110009)	Rutaceae	Tree	Stem bark, leaves	5	14.29	NE
17.	Ayin	African birch	*Anogeissus leiocarpus* (DC.) Guill. & Perr (FHI 107122)	Combretaceae	Tree	Stem bark, root, leaves	4	11.43	NE
18.	Idi	Tropical carpet grass	*Axonopus compressus* (Sw.) P.Beauv (FHI 109977)	Poaceae	Grass	Leaves	4	11.43	LC
19.	Osan-laimu or Osan agan	Lemon	*Citrus limon* (L.) Osbeck (FHI 110008)	Rutaceae	Tree	Fruit	4	11.43	LC
20.	Ponhan	Red iron wood	*Lophira alata* Banks *ex* Gaertn (FHI 109820)	Ochnaceae	Tree	Stem bark	4	11.43	VU
21.	Dongoyaro	Neem	*Azadirachta indica* A.Juss (FHI 112927)	Meliaceae	Tree	Root, leaves	4	11.43	LC
22.	Owu or Owu akese (Yoruba), Lulu fùkà (Nupe)	Cotton plant	*Gossypium barbadense* Linn (FHI 107327)	Malvaceae	Shrub	Leaves	4	11.43	NE
23.	Karandafi or Poroporo	Red sorghum	*Sorghum bicolor* (L.) Moench (FHI 109659)	Poaceae	Grass	Leaves, root	4	11.43	LC
24.	Okuuku	Giant rattan	*Ancistrophyllum* *secundiflorum* (P.Beauv.) G.Mann & H.Wendl (FHI 50908)	Arecaceae	Climber	Stem bark	3	8.57	NE
25.	June 12	Tree marigold or Mexican sunflower	*Tithonia diversifolia* (Hemsl.) A. Gray (FHI 108055)	Asteraceae	Shrub	Leaves	3	8.57	NE
26.	Emi	Shea tree	*Vitellaria paradoxa* C.F. Gaertn (FHI 107924)	Sapotaceae	Tree	Root, leaves	3	8.57	VU
27.	Ataile	Ginger	*Zingiber officinale* Roscoe (FHI 107935)	Zingiberaceae	Herb	Rhizome	3	8.57	DD
28.	Pandoro	Sausage tree	*Kigelia africana* (Lam.) Benth (FHI 107654)	Bignoniaceae	Tree	Stem bark, fruits	3	8.57	LC
29.	Arunje	Dragon’s blood tree	*Harungana madagascariensis* Lam. ex Poir (FHI 107392)	Hypericaceae	Tree	Stem bark, leaves	2	5.71	LC
30.	Gilofa or Gurofa	Guava	*Psidium guajava* Linn (FHI 110937)	Myrtaceae	Shrub	Leaves	2	5.71	LC
31.	Emi gbegiri or Akodinrin	Dry-zone cedar	*Pseudocedrela kotschyi* (Schweinf.) Harms (FHI 106873)	Meliaceae	Tree	Stem bark	2	5.71	LC
32.	Ọpẹ oyinbo	Pineapple	*Ananas comosus* (L.) Merr (FHI 58509)	Bromeliaceae	Shrub	Peel	2	5.71	NE
33.	Tude	Powder puff	*Calliandra haematocephala* Hassk (FHI 45788)	Fabaceae	Shrub	Root	2	5.71	NE
34.	Efirin	Scent basil	*Ocimum gratissimum* Linn (FHI 111995)	Lamiaceae	Herb	Leaves	2	5.71	NE
35.	Ewuro	Bitter leaf	*Vernonia amygdalina* Delile (FHI 112924)	Asteraceae	Shrub	Leaves	2	5.71	NE
36.	Ayan	African mesquite	*Prosopis africana* (Guill. & Perr.) Taub (FHI 112370)	Fabaceae	Tree	Leaves, root	2	5.71	LC
37.	Mafowokan omomi or Ahon ekun	Mountain Thistle	*Acanthus montanus* (Nees) T. Anderson (FHI 107529)	Acanthaceae	Shrub	Leaves	1	2.86	LC
38.	Akogun (Yoruba), Kwagũ̀gi (Nupe)	Dutchman’s pipe	*Aristolochia ringens* Vahl (FHI 112929)	Aristolochiaceae	Climber	Root	1	2.86	NE
39.	Gbere (Nupe)	Breadfruit	*Artocarpus altilis* (Parkinson) Fosberg (FHI 110483)	Moraceae	Tree	Root	1	2.86	NE
40.	Orombo	Sweet orange	*Citrus sinensis* (L.) Osbeck (FHI 108811)	Rutaceae	Tree	Stem bark, leaves	1	2.86	NE
41.	Agbon	Coconut palm	*Cocos nucifera* Linn (FHI 109665)	Arecaceae	Tree	Husk	1	2.86	NE
42.	Ataile pupa	Turmeric	*Curcuma longa* Linn (FHI 106920)	Zingiberaceae	Herb	Rhizome	1	2.86	DD
43.	Iya	African copaiba balsam tree	*Daniellia oliveri* (Rolfe) Hutch. & Dalziel (FHI 36952)	Fabaceae	Tree	Stem bark	1	2.86	LC
44.	Igbaluwere or Ogurobe	Splinter bean	*Entada africana* Guill. & Perr (NIPRD/H/6412)	Fabaceae	Tree	Stem bark	1	2.86	LC
45.	Ipin	Sandpaper	*Ficus exasperata* Vahl (FHI 109550)	Moraceae	Tree	Leaves	1	2.86	LC
46.	Obo	Gutta percha tree	*Ficus platyphylla* Del. Holl (FHI 78251)	Moraceae	Tree	Stem bark	1	2.86	LC
47.	Orogbo	Bitter kola	*Garcinia kola* Heckel (FHI 109481)	Clusiaceae	Tree	Stem bark	1	2.86	VU
48.	Lapalapa funfun	Bubble bush	*Jatropha curcas* Linn (FHI 109020)	Euphorbiaceae	Shrub	Leaves	1	2.86	LC
49.	Ogbesi	Pheasant-berry	*Margaritaria discoidea* (Baill.) G.L. Webster (FHI 43971)	Phyllanthaceae	Tree	Root	1	2.86	LC
50.	Ogede agbagba	Banana	*Musa paradisiaca* Linn (FHI 110122)	Musaceae	Herb	Leaves	1	2.86	NE
51.	Ogbo	African Parquetina	*Parquetina nigrescens* (Afzel.) Bullock (FHI 110044)	Apocynaceae	Shrub	Leaves	1	2.86	NE
52.	Nla	Avocado	*Persea americana* Mill (FHI 109444)	Lauraceae	Tree	Stem bark	1	2.86	LC
53.	Abafe	Wild bauhinia	*Piliostigma thonningii* (Schum.) Milne-Redh (FHI 107815)	Fabaceae	Tree	Stem bark	1	2.86	NE
54.	Iyere	Climbing black pepper	*Piper guineense* Schum. & Thonn (FHI 112922)	Piperaceae	Climber	Fruit	1	2.86	LC
55.	Sigo	Elephant’s sugarcane	*Cussonia barteri* Hochst. Ex A. Rich (UBHdt/SN/173)	Araliaceae	Tree	Leaves	1	2.86	LC
56.	Jelenubenu (Yoruba), Gayà ebá (Nupe)	Coffee senna or septic weed	*Senna occidentalis* (L.) Link (FHI 109866)	Fabaceae	Shrub	Leaves	1	2.86	LC
57.	Ajarere	Podocarpa leaf	*Senna podocarpa* (Guill. & Perr.) Lock **(**FHI 109903)	Fabaceae	Shrub	Leaves	1	2.86	NE
58.	Isekotu (Yoruba), Sàngi yèkó (Nupe)	Common wireweed	*Sida acuta* Burm.f (FHI 112276)	Malvaceae	Weed	Leaves	1	2.86	NE
59.	Opon	Cup of water	*Tetracera potatoria* Afzel. ex G. Don (FHI 105782)	Dilleniaceae	Climber	Stem bark	1	2.86	NE
60.	Aridan/Aidan	Soup perfume	*Tetrapleura tetraptera* (Schum. & Thonn.) Taub (FHI 110141)	Fabaceae	Tree	Fruit	1	2.86	LC
61.	Eeru alamo	Ethiopian or Negro pepper	*Xylopia aethiopica* (Dunal) A. Rich (FHI 108978)	Annonaceae	Tree	Stem bark	1	2.86	LC
62.	Orin ata	Senegal prickly-ash	*Zanthoxylum zanthoxyloides* (Lam.) Zepern. & Timler (NIPRD/H/7101)	Rutaceae	Tree	Stem bark	1	2.86	LC

Use value – It is an index or a number that is used in ethnobotany to quantify the importance of plant species for a specific purpose, deduced from the number of mentions by respondents

Citation frequency – It is used to calculate the percentage importance of each plant species. It is computed by dividing the number of times a specific plant species is mentioned by the total number of respondents participating in the study multiplied by 100

*Abbreviations: DD* Data Deficient, *NE* Not Evaluated, *LC* Least Concern, *VU* Vulnerable, *NT* Near Threatened, *EN* Endangered, *CR* Critically Endangered, *EW* Extinct in the Wild, *EX* Extinct

Many of the medicinal plants identified in this study are also utilised for malaria treatment in other parts of Nigeria [24, 43, 64]; and several other nations in Africa including Benin Republic [65]; Cameroon, Kenya and Namibia [66]; Ethiopia [67, 68]; Togo [69] and Zimbabwe [70]. Additionally, they have also been reported to be used for the treatment and management of a wide range of ailments and health disorders such as chronic diarrhoea, jaundice, insomnia and rheumatism [71, 72]; asthma, warts, cancer, chicken pox and gonorrhoea [23, 73]; fever, hepatitis, tuberculosis and urinary tract infections [74, 75]; cough, bronchitis, laryngitis and hoarseness of voice [76–78]; nasopharyngeal, heamorrhoids, skin infections and infertility [79]; bronchitis, stomach ache, monorrhagia, high blood pressure and diabetes [80, 81].

When compared to similar ethnobotanical surveys carried out in north-eastern [42], south-western [9, 43–45, 64] and south-eastern Nigeria [23], this present study recorded a higher number of plant taxa used for malaria treatment. Nevertheless, it recorded fewer number of plants in comparison to Oyeyemi et al. [24] in south-western Nigeria. Noteworthily, 13 new plants which have not been reported in previous ethnobotanical surveys of plants used for malaria treatment in Nigeria were identified in this study (*Acanthus montanus*, *Calliandra haematocephala*, *Pseudocedrela kotschyi*, *Vitellaria paradoxa*, *Ancistrophyllum secundiflorum*, *Waltheria indica*, *Byrsocarpus coccineus*, *Piliostigma thonningii*, *Cussonia barteri*, *Senna occidentalis*, *Tetracera potatoria*, *Ficus platyphylla* and *Entada africana*). Altogether, the data obtained in this study contribute to the concerted effort globally to record local plants and their accompanying indigenous knowledge for the benefit of the present and succeeding generations [26, 82]. Additionally, the present study adds to the existing collection of medicinal plants in Nigeria [26, 43, 45, 73, 78, 83, 84].

Our results showed that a total of 36 medicinal plant families were used as traditional antimalarial medicine (Table 1). Regarding the number of identified species, the family Fabaceae was the most dominant plant family, represented with 9 species (15%). This was followed by Rutaceae 4 species (6%), Poaceae, Meliaceae, Malvaceae and Moraceae with 3 species (5%) each. Twenty-three (63.89%) other families were represented with a single species (2%) each. The dominance of Fabaceae as the most represented family has been reported in previous ethnobotanical surveys in Nigeria [45, 73, 78, 85, 86], as well as in other African countries including Ghana [87], South Africa [88], the Democratic Republic of Congo [89], and Uganda [90]. These data suggest a richness of their local flora species and affirm the popularity of Fabaceae for their curative effectiveness.

### Habit of plants utilised for malaria treatment among the informants

**Fig. 2:**
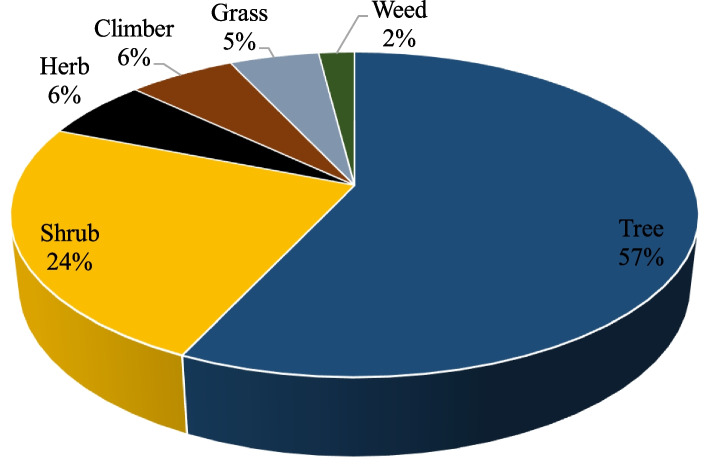
Percentage distribution of habit of medicinal plants used for malaria treatment

The inventory of 62 indigenous medicinal plants was represented by different plant habits which were dominated by woody species (81%) comprising trees (57%) and shrubs (24%) (Fig. 2). The remaining 20% of the plants were distributed among herb (6%), climber (6%), grass (5%) and weed (2%). The strong connection between the predominant local plants corresponds to the dominance of plant habit used for medicine among the TMPs [87, 91]. In comparison with herbaceous plant species, the preponderance of trees and shrubs in folk medicine is linked to their comparatively longer accessibility and persistence of the various plant parts used [92].

### Parts of the medicinal plants used for preparation of herbal medicine for malaria treatment

**Fig. 3:**
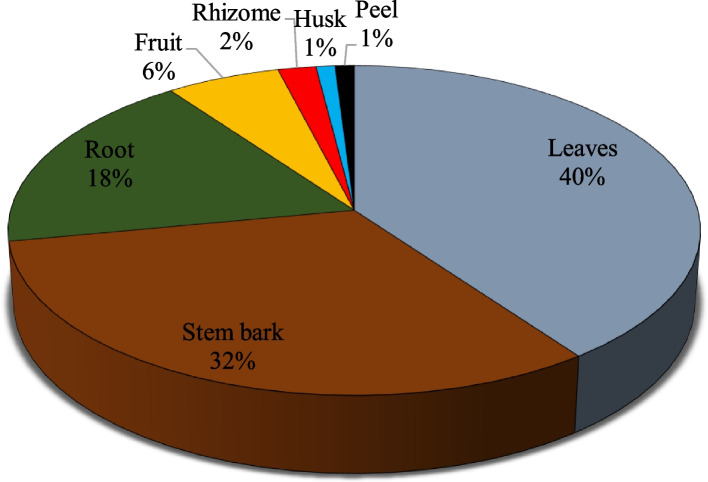
Percentage of different plant parts utilised for herbal preparation

Figure 3 showed that leaves (40%) were the most utilised medicinal plant parts for preparation of herbal medicine for malaria treatment, solely or in combination with other plant parts. This was followed by stem bark (32%), root (18%) and fruit (6%) while the least commonly used parts were rhizome (2%), husk (1%).

Plant parts are capable of accumulating different important natural phytocompounds, which offer significant pharmaceutical potentials [93]. The dominance of leaves over other plant parts in preparing herbal remedies could be linked to their being the primary site of photosynthetic and other biosynthetic activities in plants, leading to the production and accumulation of photosynthates which contain higher concentrations of bioactive molecules including alkaloids and tannins with rich medicinal properties [94–96]. Another reason could be because of the ease of handling them [97]. Again, leaves are more readily available hence, they are easily accessible and harvested in large quantities for use when compared to other parts of the plant. Harvesting leaves does not exert much strain on plant regeneration and also does not extensively harm the plants compared to the use of stem barks, roots, and/or the whole plant [98, 99]. Furthermore, from a conservation and sustainability point of view, leaves are preferred over stem barks and roots since they are not as closely linked to the survival rate of plants. So, collecting the leaves biomass within acceptable limits does not cause serious interference with the plant life.

### Traditional malaria medicine recipes, modes of preparation, routes of administration and duration of treatment

The TMPs revealed different recipes for preparing herbal medicine for malaria treatment, modes of preparation and administration, as well as duration of administration with possible side effects. Overall, 35 different polyherbal recipes were obtained from the informants. The recipes contain various plant parts including leaves, stem bark and roots, and ranged from 3 to 13 different plants used together. Although some of the plants can be used singly, earlier reports have claimed that the use of plant mixture in herbal preparation may enhance the synergism of phytochemicals of the different plants so as to elicit maximum therapeutic efficacy, and also cure several malaria-associated dysfunctions in the body [43, 90, 100].

**Fig. 4:**
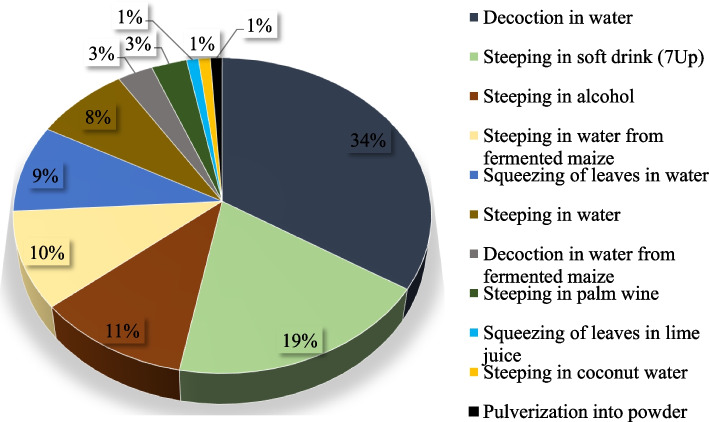
Distribution of traditional modes of preparation of herbal medicine

Of the 11 different traditional methods reported to be used for preparation of malaria therapy by the TMPs, decoction in water (34%) was the most preferred method of extracting the bioactive ingredients from the plants (Fig. 4). The different components of the recipes are uniquely arranged with the stem bark and/or root usually cut into smaller pieces and placed at the bottom of the pots followed by the leaves, fruits and other plant parts. Other modes of preparation included steeping (infusion) in non-caffeinated soft drink (7up) (19%), steeping in alcohol “*ogogoro*” (11%) and steeping in water from fermented maize “*omi ogi*” or “*omi idun*” (10%). The least preferred methods were squeezing of leaves in lime juice (1%), steeping in coconut water (1%) and pulverization into powder (1%). The choice of decoction and steeping as the most preferred traditional methods of preparing malaria herbal remedies is consistent with earlier reports [101–104]. The main reason why these modes of preparation are widely utilised by the TMPs could be because they are simple, easy to handle and cheap [78]. In addition, decoction enhances extraction of bioactive ingredients from the plant parts much more in comparison to cold extraction. However, several factors including boiling duration, amount of solvent and plant material used may differ which could possibly affect the potency of the herbal preparations [61]. Also, both decoction and steeping do not provide long shelf life for the herbal preparations and as a result, the medicinal plants would need to be continuously harvested thereby putting them under considerable pressure that may lead to overexploitation.

**Fig. 5:**
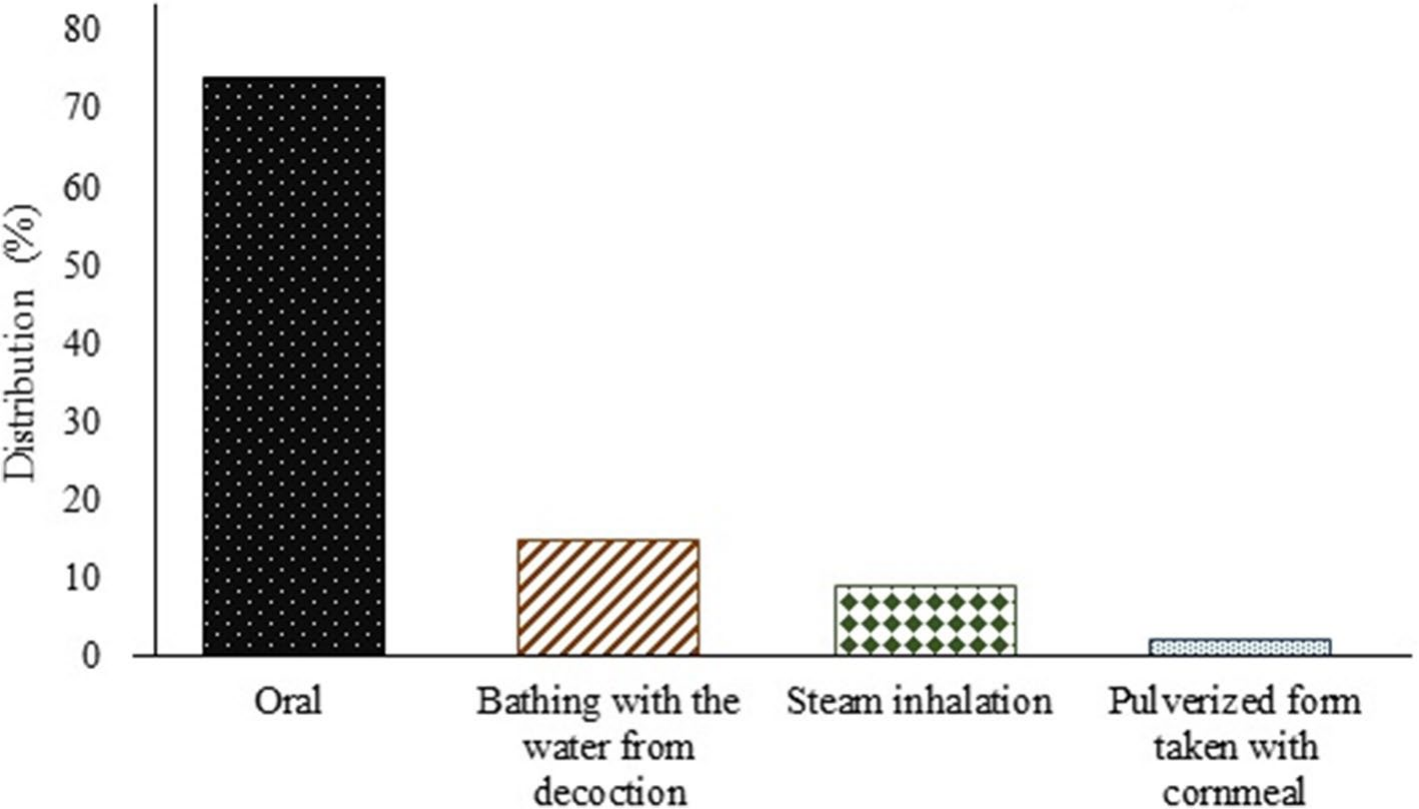
Distribution of different modes of administration of herbal preparation

Regarding administration of herbal preparation, oral administration (74%), bathing with water from decoction (15%), steam inhalation (9%) and pulverized form of plant parts taken with cornmeal (2%) were reported to be the routes of administration (Fig. 5). In this report, oral administration was considered to be the main route of administering herbal preparations, consistent with previous investigations [63, 105–107].

### Pharmacological relevance of the extracts and phytocompounds isolated from the identified plants

As a result of the growing interest in phytoremediation as an alternative therapeutic strategy for combating malaria, many bioactive compounds have been extracted, isolated and characterised from medicinal plants using different methods including chromatographic and spectrophotometric techniques [108–111]. Interestingly, most of the medicinal plants recorded in this study have been demonstrated through in vitro and/or in vivo approaches to possess antimalarial activity (Table 2), supporting the traditional use of these plants for malaria treatment in the region. Over 50 compounds (Table 3), with varying antimalarial activities, have been isolated from some of the plants documented in this study including *E. chlorantha*, *V. amygdalina*, *M*. *lucida*, *A. occidentale*, *H. madagascariensis*, *K. africana*, *G. kola*, *A. indica*, *X. aethiopica*, *C. papaya* and *P. americana*. The following is a brief description of some of the medicinal plants and their constituents previously investigated for their antimalarial property.

**Table 2:** Scientific evaluation of the antimalarial properties of the identified medicinal plants and their phytocompounds

S/n	Name of plant	Parts of plant used	Extraction solvent	Antimalarial activity of extracts and parasite suppression rate	IC_50_	Isolated compounds with antimalarial activity
1.	*E. chlorantha*	Stem bark	Ethanol, water, methanol, dichloromethane, hexane	In vitro Boyom et al. [112] Very good to moderate activity *P. falciparum* W2 strain	0.68–14.72 µg/ml IC_50_	Jatrorrhizine, palmatine and berberine In vitro and in vivo [113]
2.	*A. indica*	Leaf Stem bark	Hydroethanol Dichloromethane	In vivo [114] Suppression of 49.75 ± 3.64 to 69.28 ± 1.36% against *P. berghei* NK65 at 75–300 mg/kg In vitro [115] Very good activity against *P. falciparum* W2 strain	4.7 µg/ml IC_50_	Nimbolide In vitro and in vivo [116] Gedunin In vitro [117] Azadirachtin In vitro [118]
3.	*M. indica*	Stem bark	Ethanol	In vivo [119] Suppression of 76.9 ± 0.02% against *P. berghei* NK65 strain at 1,000 mg/kg		
4.	*M. lucida*	Leaf Root Leaf	Hydroethanol (50:50) Aqueous Methanol	In vivo [120] Suppression of 96.24% at 200 and 400 mg/kg In vivo [119] Suppression of 60.9 ± 0.01% against *P. berghei* NK65 strain at 1,200 mg/kg In vitro [121] Excellent activity against *P. falciparum*	25 nM IC_50_	Digitolutein, rubiadin 1-methyl ether and damnacanthal In vitro [122] Ursolic acid and Oleanolic acid In vitro and in vivo [123]
5.	*X. aethiopica*	Seed	Aqueous	In vitro [124] Moderate activity against *P. falciparum* 3D7 strain	14 µg/ml IC_50_	Xylopic acid In vivo [125]
6.	*A. occidentale*	Apple	Ethanol	In vitro [126] Poor activity against *P. falciparum* D6 strain	0.577 g/ml IC_50_	Anacardic acid In vitro [127] Cardol triene 1 and 2-methylcardol triene 4 In vitro [126]
7.	*C. limon*	Leaf	Dichloromethane	In vitro [128] Good activity against *P. falciparum* D10 strain	5.0 µg/ml IC_50_	
8.	*C. citratus*	Leaf	Dried and ground into powder	In vivo [129] Suppression effect of 99.89% at 1,600 mg/kg against *P. berghei* ANKA		
9.	*L. inermis*	Leaf	Dichloromethane:methanol (1:1)	In vivo [84] Suppression effect of 68.20% at 250 mg/kg against *P. berghei* ANKA strain		
10.	*A. boonei*	Leaf Stem bark	Ethanol Ethanol	In vivo [130] Suppression effect of 74.9% at 400 mg/kg against *P. berghei* NK65 strain In vivo [131] Suppression effect of 75% at 400 mg/kg against *P. berghei* NK65 strain		
11.	*N. latifolia*	Leaf	Ethanol	In vivo [132] Suppression effect of 60.63% at 500 mg/kg against *P. berghei*		
12.	*A. altilis*	Leaf and stem bark	Ethanol	In vivo and in vitro [133] Very active against *P. berghei* ANKA at ED_50_ value of 0.82 mg/kg Very good activity against *P. falciparum* 3D7 strain	1.32 μg/ml IC_50_	1-(2,4-dihydroxy phenyl)-3-[8-hydroxy-2-methyl-2-(4-methyl-3-pentenyl)-2H-1-benzopyran-5-yl]-1-propanone In vitro [134]
13.	*C. papaya*	Leaf Leaf	n-Hexane, ethyl acetate and dichloromethane Ethyl acetate	In vitro [135] Very good to moderate activity against *P. falciparum* 3D7 and Dd2 strains In vitro [136] Very good activity against *P. falciparum* D10 strain	1.52–20.32 g/ml IC_50_ 2.96 µg/ml IC_50_	Carpaine In vitro [135] Linoleic and linolenic acids In vitro [136]
14.	*C. longa*	Leaf	Ethanol	In vivo [130] Suppression effect of 78.4% at 400 mg/kg against *P. berghei* NK65 strain		
15.	*V. amygdalina*	Leaf Leaf Leaf	Ethanol Ethanol Aqueous	In vitro and in vivo [137] Good activity against *P. falciparum* 3D7 Suppression of 82.3% at 1,000 mg/kg against *P. berghei* ANKA In vivo [138] Suppression of 67% at 1,000 mg/kg against *P. berghei* In vivo [139] Suppression of 63% at 125 mg/kg against *P. berghei* ANKA	9.83 µg/ml IC_50_	Vernonioside A1, A2, A3, A4, B1, vernodalin, vernodalol, vernolide and hydroxyvernolide In vitro [140]
16.	*T. cacao*	Leaf	Aqueous	In vivo [141] Suppression effect of 79.19% at 400 mg/kg against *P. berghei* ANKA strain		
17.	*L. alata*	Leaf	Hexane	In vitro and in vivo [142] Very good activity against *P. falciparum* NF54 and K1 strains Suppression effect of 74.45% at 500 mg/kg against *P. berghei* NK65	2.5 µg/ml IC_50_	
18.	*C. nucifera*	Husk fibre	Ethyl acetate	In vitro and in vivo [143] Good activity against *P. falciparum* W2 strain Suppression effect of 98.6% at 125 mg/Kg against *P. berghei* NK65	10.94 μg/ml IC_50_	
19.	*H. madagascariensis*	Leaf Stem bark	Methanol Ethanol	In vitro [144] Weak activity against *P. falciparum* D6 strain In vitro and in vivo [145] Poor activity against *P. falciparum* Suppression of 28.6–44.8% at 20–80 mg/kg against *P. yoelii nigeriensis* N67 strain	39.07 ± 1.57 μg/ml 0.052–0.517 g/ml IC_50_	Bazouanthrone, feruginin, harunganin, harunganol A and harunganol B In vitro [146]
20.	*K. africana*	Leaf, root, stem bark and fruit Stem bark	n-Hexane, dichloromethane, ethyl acetate, n-butanol and methanol Ethyl Acetate	In vivo [9] Suppression effect of 70.33- 84.73% at 500 mg/kg against *P. berghei* NK65 In vitro [147] Moderate activity against *P. falciparum* W2 strain	11.15 μg/ml IC_50_	*p*-hydroxycinnamic acid, specicoside, 2β,3β,19α-trihydroxy-urs-12-en28-oic acid and atranorin In vitro [148]
21.	*S. acuta*	Leaf Leaf	Methanol Ethanol	In vivo [149] Suppression effect of 64.64% at 600 mg/kg against *P. berghei* NK 65 strain In vivo [150] Suppression effect of 97.38% at 600 mg/kg against *P. berghei* ANKA-65 strain		Cryptolepine In vitro [151]
22.	*Z. zanthoxyloides*	Leaf Root	Methanol Aqueous	In vivo [152] Suppression effect of 95.95% at 600 mg/kg against *P. berghei* ANKA-65 strain In vitro [153] Very good activity against *P. falciparum* strain 3D7	4.9 μg/mL IC_50_	Fagaronine In vitro [153, 154]
23.	*T. diversifolia*	Aerial part	Ethanol	In vivo [155] Suppression effect of 82.55% at 400 mg/kg against *P. berghei var Anka 1* strain		Tagitinin C In vitro [156]
24.	*G. kola*	Stem bark Seed	Ethanol and petroleum ether n-Hexane, dichloromethane and methanol	In vitro [157] Very good activity against *P. falciparum* In vivo and in vitro [158] Moderate activity against *P. falciparum* D10 strain	< 3 µg/ml IC_50_ 10.59–25.65 µg/mL IC_50_	Kolaviron In vivo [159] I-4′,II-4′,I-5,II-5,I-7,II-7-hexahydroxy-I-3,II-8-biflavanone, II-3,I-4′,II-4’,I-5,II-5,I-7,II-7-heptahydroxy-I-3,II8-biflavanone and II-3, 3′,I-4′,II-4′,I-5,II-5,I-7, II-7-octahydroxy-I-3,II-8-biflavanone In vitro and in vivo [160]
25.	*C. aurantifolia*	Leaf	Methanol	In vivo [161] Suppression effect of 75.66% at 320 mg/kg against *P. berghei* NK65 strain		
26.	*P. guajava*	Leaf Leaf	Ethanol Aqueous	In vivo [162] Suppression effect of 58.9 and 67.4% at 600 mg/kg against *P. berghei* ANKA strain and *P. chabaudi chabaudi* In vitro [163] Good activity against *P. falciparum* K1 strain	5.46 µg/ml IC_50_	
27.	*K. ivorensis*	Stem bark	Aqueous	In vivo [164] Suppression effect of 35.5% at 400 mg/kg against *P. berghei* ANKA strain		
28.	*A. nilotica*	Leaf, pods and bark Twigs	Hydroethanol Dichloromethane/ methanol	In vitro [165] Very good activity against *P. falciparum* 3D7 strain In vitro [166] Moderate activity against *P. falciparum* D10 strain	1.29, 4.16 and 4.28 μg/ml IC_50_ 13 µg/ml IC_50_	
29.	*B. coccineus*	Leaf	Ethanol	In vivo [167] Suppression effect of 92.3% at 400 mg/kg against *P. berghei berghei*		
30.	*W. indica*	Aerial parts	Aqueous and dichloromethanol:methanol (1:1)	In vitro [166] Poor activity against *P. falciparum* D10 strain	> 100 μg/ml IC_50_	
31.	*A. leiocarpus*	Stem bark	Methanol, water, butanol and ethyl acetate	In vitro [168] Good to moderate activity against *P. falciparum* 3D7 and K1	10.94–13.77 µg/ml IC_50_	Castalagin, ellagic acid, flavogallonic acid and punicalagin In vitro [168]
32.	*A. compressus*	Leaf	Aqueous	In vivo [169] Suppression effect of 85.7% at 62.5 mg/kg against *P. berghei* NK65		
33.	*V. paradoxa*	Leaves and stem bark Stem bark	70% aqueous methanol Dichloromethane	In vitro [170] Weak to very weak activity against *P. falciparum* 3D7 strain In vitro [171] Weak activity against *P. falciparum* 3D7 strain	39 and 66 µg/ml IC_50_ 43.94 ± 13.44 µg/ml IC_50_	
34.	*Z. officinale*	Root	Hydromethanol	In vivo [172] Suppression effect of 32.83 ± 1.03% at 1000 mg/kg against *P. berghei* ANKA strain		
35.	*P. kotschyi*	Leaf	Ethanol	In vivo [173] Suppression effect of 90% at 400 mg/kg against *P. berghei* NK65 strain		Kotschyins A-C, 7-deacetylgedunin and 7-deacetyl-7-oxogedunin In vitro [174]
36.	*A. comosus*	Leaf	Ethanol	In vitro [175] Very weak activity against *P. falciparum* 3D7 and INDO strains	> 100 µg/ml IC_50_	
37.	*P. americana*	Leaf	Methanol, n-hexane, ethyl acetate, methanol and aqueous	In vivo [176] Suppression effect of 55.00 ± 0.06 to 64.01 ± 0.08% at 200–400 mg/kg against *P. berghei*		1, 2, 4-trihydroxyheptadec-16-ene and 1, 2, 4, 15-tetrahydroxyheptadecane-6, 16-diene In vitro [177]
38.	*O. gratissimum*	Fresh leaf Leaf Leaf and twig Leaf	Aqueous (Hydrodistillation) Aqueous Dichloromethane Methanol	In vivo [178] Suppression effect of 77.8% at 500 mg/kg against *P. berghei* ANKA strain In vitro [163] Good activity against *P. falciparum* K1 strain In vitro [179] Good activity against *P. falciparum* D6 and W2 strains In vitro [180] Good activity against *P. falciparum* D6 and W2 strains	7.25 µg/ml IC_50_ 8.616 µg/ml IC_50_ 5.9 µg/ml IC_50_	
39.	*P. africana*	Stem bark	Methanol, aqueous, butanol and ethyl acetate	In vitro [168] Moderate activity against *P. falciparum* 3D7 and K1	14.97–15.28 µg/ml IC_50_	
40.	*C. sinensis*	Stem Rind of ripe fruit	Ethanol Petroleum ether and methanol	In vivo [181] Suppression effect of 53.27% at 700 mg/kg against *P. berghei* ANKA strain In vitro [182] Very weak activity against *P. falciparum* FCK 2 strain	51.06–53.61 µg/ml IC_50_	
41.	*D. oliveri*	Leaf Stem bark	Methanol Methanol, aqueous, butanol and ethyl acetate	In vivo [183] Suppression effect of 87% at 700 mg/kg against *P. berghei* In vitro [168] Weak activity against *P. falciparum* 3D7 and K1 strains	23.14–32.97 µg/ml IC_50_	
42.	*F. exasperata*	Leaf	Ethanol	In vivo [184] Suppression effect of 82% at 400 mg/kg against *P. berghei* NK65 strain		
43.	*F. platyphylla*	Stem bark	Ethanol	In vivo [185] Suppression effect of 43.50% at 300 mg/kg against *P. berghei*		
44.	*J. curcas*	Leaf	Ethyl acetate, hexane and methanol	In vitro [158] Good to moderate activity against *P. falciparum* K1 and NF54 strains	2.39 ± 0.54- 31.09 ± 4.36 µg/ml IC_50_	
45.	*M. discoidea*	Leaf	Aqueous, ethanol and methanol	In vitro [115] Moderate to weak activity against *P. falciparum* K1 and NF54 strains	13.60 ± 1.80- 43.61 ± 0.92 µg/ml IC_50_	
46.	*P. nigrescens*	Leaf	Aqueous	In vivo [186] Suppression effect of 86% at 150 mg/kg against *P. berghei* NK65		
47.	*P. thonningii*	Leaf	Ethanol	In vivo [187] Suppression effect of 91.94% at 400 mg/kg against *P. berghei* NK65		
48.	*P. guineense*	Leaf	Ethanol	In vivo [188] Suppression effect of 62.69% at 600 mg/kg against *P. berghei*

**Table 3:** Phytocompounds with antimalarial activities isolated from some of the indigenous medicinal plants used for malaria treatment

S/n	Name of plant	Isolated phytocompounds with antimalarial activity	Structure
1.	*A. occidentale*	Anacardic acid [127]	
		Cardol triene 1 [126]	
		2-methylcardol triene 4 [126]	
2.	*A. indica*	Gedunin [117]	
		Nimbolide [116]	
		Azadirachtin [118]	
3.	*C. papaya*	Carpaine [135]	
		Linoleic acid [136]	
		Linolenic acid [136]	
4.	*E. chlorantha*	Jatrorrhizine [113]	
		Berberine [113]	
		Palmatine [113]	
5.	*M. lucida*	Digitolutein [122]	
		Rubiadin 1-methyl ether [122]	
		Damnacanthal [122]	
		Ursolic acid [123]	
		Oleanolic acid [123]	
6.	*P. americana*	1, 2, 4-trihydroxyheptadec-16-ene [177]	
		1, 2, 4, 15-tetrahydroxyheptadecane-6, 16-diene [177]	
7.	*V. amygdalina*	Vernonioside A1: R_1_ = beta-OH, R_2_ = H [140] Vernonioside A2: R_1_ = alpha-OH, R_2_ = H [140] Vernonioside A3: R_1_ = O, R_2_ = H [140] Vernonioside B1: R_1_ = H, R_2_ = OH [140]	Glc
		Vernonioside A4 [140]	
		Vernodalin [140]	
		Vernodalol [140]	
		Vernolide [140]	
		Hydroxyvernolide [140]	
8.	*G. kola*	Kolaviron [159]	
		I-4′,II-4′,I-5,II-5,I-7,II-7-hexahydroxy-I-3,II-8-biflavanone: R^1^ = H, R^2^ = H [160] II-3,I-4′,II-4’,I-5,II-5,I-7,II-7-heptahydroxy-I-3,II8-biflavanone: R^1^ = H, R^2^ = OH [160] II-3, 3′,I-4′,II-4′,I-5,II-5,I-7, II-7-octahydroxy-I-3,II-8-biflavanone: R^1^ = OH, R^2^ = OH [160]	
9.	*H. madagascariensis*	Bazouanthrone: R_1_ = Prenyl, R_2_ = Prenyl, R_3_ = OH, R_4_ = H [146] Feruginin: R_1_ = H, R_2_ = Prenyl, R_3_ = H, R_4_ = Prenyl [146] Harunganin: R_1_ = H, R_2_ = Prenyl, R_3_ = Prenyl, R_4_ = H [146] Harunganol A: R_1_ = H, R_2_ = H, R_3_ = Prenyl, R_4_ = Prenyl [146] Harunganol B: R_1_ = H, R_2_ = H, R_3_ = Prenyl, R_4_ = H [146]	
10.	*K. africana*	Specicoside [148]	
		Atranorin [148]	
		2β,3β,19α-trihydroxy-urs-12-en28-oic acid [148]	
		*p*-Hydroxycinnamic acid [148]	
11.	*X. aethiopica*	Xylopic acid [125]	

*Alstonia boonei* De Wild is an evergreen tree, that is widely distributed in the tropical and rain forests zones of West and Central Africa. The plant is listed in African pharmacopoeia and is commonly applied in Nigeria for the treatment of a variety of ailments including malaria, chronic diarrhoea, insomnia, jaundice and rheumatic pains [72, 189]. Herbal tincture and decoction of the stem bark of the plant is also used as an effective antidote against scorpion or snake poison, as well as for inducing lactation and expelling retained products of afterbirth when administered to women [190]. Infected mice with the chloroquine-sensitive *Plasmodium berghei* NK65 parasite were used to study the in vivo anti-plasmodial effects (suppressive, curative and prophylactic models) of the ethanolic stem bark extract of the plant. The extract demonstrated significant (*p* < 0.05) suppressive (46.43–75%), curative (61.02–81.36%) and prophylactic (34.83–60.67%) antiplasmodial effects against the parasite in a dose-dependent (100, 200 and 400 mg/kg) manner [131]. Also, Agbedahunsi et al. [130] investigated the in vivo chemosuppressive effect of the ethanolic leaf extract of the plant in *P. berghei*–infected mice and their results revealed a chemosuppression of 0.2–74.8%, dose-dependently (12.5–400 mg/kg).

*Anacardium occidentale* L. is a nut tree crop widely cultivated in tropical regions of the world including Nigeria. Decoction of the leaves and stem bark have been reported to be used in ethnomedicine for malaria treatment and management of severe diarrhoea in Nigeria [64, 71]. The in vitro antiplasmodial investigation of ethanol extract of cashew apple demonstrated significant activity with an IC_50_ of 0.577 g/ml [126]. Besides, the phytocompounds cardol triene 1 (IC_50_ = 5.69 M) and 2-methylcardol triene 4 (IC_50_ = 5.39 M) isolated from the plant showed significant antimalarial activity in vitro when evaluated against *P. falciparum* D6 strain [126]. Additionally, anacardic acid, another phytocompound from *A. occidentale*, was demonstrated to possess antiplasmodial effect [127]. The compound interrupted the parasite’s transcription process by inhibiting *P. falciparum* histone acetyltransferase (PfGCN5) activity.

*Azadirachta indica* A. Juss. is a tropical and subtropical plant which is utilised for various medicinal purposes. In the tropics, the plant is used as traditional remedies for the treatment of malaria [43, 46]. Several studies have shown the seed kernel, leaf and stem bark extracts to possess antimalarial properties [191–193]. In an in vitro study, Hout et al. [194] reported a good activity (4.7 µg/ml IC_50_) of dichloromethane stem bark extract of *A. indica* against chloroquine resistant *P. falciparum* W2 strain. Tepongning et al. [114] reported that *P. berghei*-infected BALB/c mice treated with hydroethanolic leaf extract of the plant showed a significant (*p* ≤ 0.001) reduction of parasitaemia ranging from 49.75 ± 3.64 to 69.28 ± 1.36% dose-dependently. The antimalarial efficacy of tablet suspension of the leaf and bark of the plant were assessed on *P. yoelli nigeriensis*-infected mice. The tablet suspensions demonstrated high preventive to moderate suppressive and weak curative schizonticidal effects, respectively [195]. Furthermore, the in vitro antimalarial activities of various phytocompounds such as nimbolide, gedunin and azadirachtin isolated from *A. indica* have been reported [116–118].

*Carica papaya* L. is commonly administered in traditional settings for the treatment of malaria and various other maladies including cancer, asthma, warts, jaundice and malaria [23]. Using bioassay-guided fractions and dichloromethane extract, Teng et al. [135] evaluated the antimalarial activity of *C. papaya* leaf extracts in vitro against *P. falciparum* 3D7 and Dd2 strains. The hexane extract was the most potent of the extracts obtained from *C. papaya* leaves; it had an IC_50_ of 3.43 ± 0.41 and 1.52 ± 0.003 g/ml against the 3D7 and Dd2 strains, respectively. This was followed by dichloromethane leaf extract which had an IC_50_ of 7.67 ± 1.9 and 4.50 ± 0.17 g/ml against the 3D7 and Dd2 strains, respectively. The ethyl acetate leaf extract was moderately potent against the *P. falciparum* 3D7 strain, with an IC_50_ of 20.32 ± 3.5 g/mL, while the leaf juice at a dilution of 1/64 inhibited its development by 50%. In another study, Melariri et al. [136] demonstrated the antiplasmodial activity in vitro of the leaf extract of the plant. Their result showed that ethyl acetate crude extract had a very good activity with an IC_50_ of 2.96 µg/ml against *P. falciparum* D10 strain. The phytocompound carpaine isolated from *C. papaya* exhibited very good activity against *P. falciparum* 3D7 and Dd2 strains with IC_50_ of 2.01 ± 0.18 μg/ml (4.21 µM) and 2.19 ± 0.60 μg/ml (4.57 µM) against 3D7 and Dd2 strains, respectively [135]. Furthermore, both linoleic and linolenic acids isolated from the ethyl acetate leaf fraction of the plant showed good activity with IC_50_ of 6.88 μg/ml and 3.58 μg/ml against *P. falciparum* chloroquine-sensitive D10 and chloroquine-resistant DD2 strains, respectively [136].

*Daniella oliveri* (Rolfe) Hutch. & Dalziel grows predominantly in some parts of South America and Africa. In Nigeria, the plant is used in traditional medicine for the treatment of breast tumours, abscesses, and vestibule vagina fistula [196]. Also, herbal preparations with the leaves and stem bark are used for the treatment of gastrointestinal disorders, diabetes and diarrhoea, as well as malaria [197, 198]. The antiplasmodial effect of the methanol, aqueous, butanol and ethyl acetate stem bark extract revealed weak activity (IC_50_ of 23.14–32.97 µg/ml) against *P. falciparum* 3D7 and K1 strains [168]. But a suppression effect of 87% against *P. berghei* was recorded, though at a higher dose of 700 mg/kg [183].

*Enantia chlorantha* Oliv. is widely distributed along the coastal regions of West and Central Africa including Nigeria where it is utilised in traditional medicine for the treatment and management of several health disorders including urinary tract infections, jaundice, malaria, fever, tuberculosis and hepatitis [74, 75]. A decoction of 500 g of stem bark of the plant in 3 L of water for 20 min, taken orally, has been reported to treat malaria symptoms, aches, wounds, fever, and chills [199]. Boyom et al. [112] investigated the in vitro antiplasmodial activity of solvent fractions and ethanolic crude extract of *E. chlorantha* stem bark against *P. falciparum* W2 strain, and they reported a good activity at IC_50_ of 0.68 to 14.72 µg/ml. Also, the antimalarial activities of the protoberberine alkaloids compounds – jatrorrhizine, berberine and palmatine isolated from *E. chlorantha* have been tested both in vitro against *P. falciparum* and in vivo against *P. berghei*. They showed potency similar to that of quinine in vitro however, none of the compounds was active in vivo [113].

*Ficus platyphylla* Del. Holl is a deciduous heavily branched tree that is distributed widely throughout the savannah region of West African coast. In folk medicine, decoction of the seeds, leaves and stem bark of *F. platyphylla* is taken as fertility enhancement in Nigeria [200]. It is also used for the management of epilepsy, psychosis [201], and tuberculosis [202]. In Burkina Faso, the stem bark of the plant is used traditionally for malaria treatment [203]. In vivo antiplasmodial activity of the ethanolic stem bark extract of the plant suppressed malaria dose-dependently in *P. berghei*-infected mice, by 43.50% at the highest dose of 300 mg/kg [185]. Additionally, treatment with the plant extract prevented severe reduction in packed-cell volume in the infected mice revealing its capacity to remedy anaemic conditions.

*Garcinia kola* Heckel is a flowering plant widely distributed in the tropical rain forest region of West and Central Africa, and it is largely valued for its nuts. The seeds, commonly known as “bitter kola”, are edible and are usually chewed as an adjuvant to the true kola (*Cola nitida* and *C. accuminata*). In traditional medicine, preparations with different parts of the plant including the seeds, leaves and stem bark are used extensively as purgatives, aphrodisiac, as well as for the treatment of diarrhoea, liver diseases, cough, hoarseness of voice, laryngitis and bronchitis [76, 77, 204]. In vitro and in vivo antimalarial studies have been carried out on the plant. The results from Tona et al. [157] revealed that the ethanolic extract of the stem bark and its petroleum ether fraction showed very good antiplasmodial activities with IC_50_ values of < 3 µg/ml in vitro. In another study by Ujomu et al. [158], it was observed that n-hexane, dichloromethane and methanol extracts of the seeds of *G. kola* were active in vitro against chloroquine-sensitive *P. falciparum* D10 strain (10.59–26 µg/mL IC_50_). They also reported that the n-hexane extract reduced parasitaemia in *P*. *berghei*-infected mice by 70% at 400 mg/kg, prolonging survival of the animals. Three biflavanones (I-4′,II-4′,I-5,II-5,I-7,II-7-hexahydroxy-I-3,II-8-biflavanone, II-3,I-4′,II-4’,I-5,II-5,I-7,II-7-heptahydroxy-I-3,II8-biflavanone and II-3, 3′,I-4′,II-4′,I-5,II-5,I-7, II-7-octahydroxy-I-3,II-8-biflavanone) isolated from *G. kola* demonstrated potent inhibitory activity in vitro against *P. falciparum* proliferation and against *P. berghei *in vivo [160]. I-4′,II-4′,I-5,II-5,I-7,II-7-hexahydroxy-I-3,II-8-biflavanone exhibited the strongest in vitro antimalarial potency on *P. falciparum* with an IC_50_ of 0.16 μM. In the in vivo antimalarial assay in *P. berghei*-infection in mice, I-4′,II-4′,I-5,II-5,I-7,II-7-hexahydroxy-I-3,II-8-biflavanone was found to exhibit antimalarial effect with an ED_50_ of about 100 mg/kg following oral treatment. I-4′,II-4′,I-5,II-5,I-7,II-7-hexahydroxy-I-3,II-8-biflavanone was also found to increase the average life span of the infected mice significantly when compared to that of the control (*p* < 0.01).

*Harungana madagascariensis* Lam. ex Poir. is found in tropical Africa with wide distribution in areas where annual rainfall is above 1300 mm. In traditional medicine, preparations from different parts of the plant including the stem bark and leaves are used in the treatment of urogenital infections, chest pain, river blindness, hepatitis, toothache, dysmenorrhea, asthma, and malaria [205]. Iwalewa et al. [145] evaluated the in vitro and in vivo antimalarial activity of the stem bark extract of *H. madagascariensis*. The IC_50_ of the ethanolic extract of the plant on *P. falciparum* was between 0.052 and 0.517 μg/ml as against the standard drugs artemether (0.021 g/ml) and chloroquine (0.0412 g/ml). The activities of the extract in an in vivo study on *P. yoelii nigeriensis* were between 28.6–44.8 and 30.2–78.2% at 20–80 mg/kg in both suppressive and prophylactic assays, respectively, in comparison to chloroquine (70.6%) and pyrimethamine (43.3%). However, in the curative test, only 80 mg/kg of the extract decreased the level of parasitaemia in comparison to the standard drug chloroquine. In a similar report, the methanolic leaf extract of the plant showed weak in vitro activity (39.07 ± 1.57 μg/ml) against *P. falciparum* D6 strain and moderate chemosuppression of parasitaemia (53.13%) [144]. The aqueous leaf extract was inactive in vitro but showed high chemosuppression of parasitaemia (88.04%). Lenta et al. [146] evaluated the antiplasmodial activity of compounds harunganin, harunganol A, harunganol B, feruginin and bazouanthrone isolated from *H. madagascariensis* against *P. falciparum* W2 strain. All the compounds showed varying activities against the malaria parasite with bazouanthrone being the most potent (IC_50_ = 1.80 μM).

*Jatropha curcas* Linn. is a semi-evergreen shrub found in abundance in Mexico, northeastern part of South America as well as in some tropical and sub-tropical regions in Asia and Africa. Herbal preparations of the stem, leaves and seeds are widely used for various medicinal purposes in traditional settings in Africa. Decoction of the leaves is utilised to treat cough while the seed oil is used for treating several skin diseases and soothing rheumatic pain. Decoction of the leaves is also applied as an antiseptic after child delivery [206]. Moreover, the seeds are used as purgative and laxative, and for the treatment of helminthic infections, paralysis, ascites, and gouts [207]. Furthermore, drops of diluted twig-sap are administered orally to babies affected by tetanus [208]. An in vitro study of the ethyl acetate, hexane and methanol leaf extract of the plant leaves demonstrated good to moderate antiplasmodial activity (2.39 ± 0.54- 31.09 ± 4.36 µg/ml IC_50_) against *P. falciparum* K1 and NF54 strains [209].

*Kigelia africana* (Lam.) Benth. is a monospecific genus under the family Bignoniaceae which grows along watercourses and in riverine areas in West, Central and South Africa [210]. Different parts of the plant are utilised in traditional medicine for the treatment of various ailments including rheumatism, heamorrhoids, nasopharyngeal, skin infections, malaria and for fertility enhancement [79, 211]. Imran et al. [9] evaluated the extracts of the leaf, stem bark, fruit and root of *K. africana* in a 4-day antiplasmodial test in *P. berghei*-infected mice, they all showed dose-dependent chemosuppressive activity at the three administered doses of 125, 250 and 500 mg/kg, respectively. The stem bark extract exhibited the highest chemosuppressive activity of 84.73%, followed by the root with 78.06% and then the leaf (72.94%) while the fruit had the least chemosuppressive activity of 70.33% at the maximum dose of 500 mg/kg compared to the standard drug chloroquine with 93.99% chemosuppression of parasitaemia. They also evaluated the antiplasmodial effect of different solvent fractions of the extract: the chemosuppressive activity of the ethyl acetate, n-butanol, and methanol fractions were 42.61, 56.05 and 69.94%, respectively in comparison with the positive control (chloroquine) which had chemosuppression of 86.17%. Zofou et al. [148] isolated the phytocompounds *p*-hydroxycinnamic acid, specicoside, 2β,3β,19α-trihydroxy-urs-12-en28-oic acid, and atranorin from the stem bark of the plant and evaluated them against the multidrug-resistant W2mef strain of *P. falciparum*. Three of the four compounds tested showed significant activity against W2mef: specicoside (IC_50_ = 1.02 ± 0.17 μM), 2β,3β,19α-trihydroxy-urs-12-en28-oic acid (IC_50_ = 1.86 ± 0.15 μM), and atranorin (IC_50_ = 1.78 ± 0.18 μM) while on the other hand, *p*-hydroxycinnamic acid showed a moderate activity (IC_50_ = 12.89 ± 0.87 μM).

*Margaritaria discoidea* (Baill.) G.L. Webster is a tree that can grow up to 30 m in height. The plant abounds in Senegal, Cameroun and other parts of tropical Africa. The stem bark of the plant is used in folk medicine to treat malaria and helminthic infections in Cote d’Ivoire [212]. It is also utilised for the treatment of onchocerciasis in Cameroon [213]. Additionally, the decoction of the stem bark is used in the Republic of the Congo to relieve stomach discomfort, and facilitate delivery during parturition [214]. The aqueous, ethanol and methanol leaf extracts of *M. discoidea* were assessed for their in vitro antiplasmodial activity against chloroquine sensitive (NF54) and multi-resistant (K1) strains of *P. falciparum* [115]. Moderate to weak activity (13.60 ± 1.80- 43.61 ± 0.92 µg/ml IC_50_) against the K1 and NF54 strains was reported.

*Morinda lucida* Benth is a small to medium-sized tree which grows in grassland, forests and occasionally in regularly flooded areas. Decoctions and infusions of the leaves, stem bark and root of the plant are utilised as remedies in traditional settings against trypanosomiasis, feverish condition during child delivery, yellow fever, and malaria [215]. The antimalarial effects of extracts of different parts of *M. lucida* have been demonstrated. Olasehinde et al. [121] reported the antiplasmodial effect in vitro of the methanolic extract of *M. lucida* leaf. An excellent activity against *P. falciparum* was recorded at IC_50_ of 25 nM*.* Treatment of *P. berghei*-infected mice with 200 and 400 mg/kg hydroethanolic (50:50) leaf extract of the plant significantly (*p* > 0.05) reduced the level of parasitaemia (96.24%), but was slightly lower than that recorded for the standard compound chloroquine (100%) [120]. In a similar manner, decoction of *M. lucida* root in distilled water demonstrated significant (*p* < 0.05) chemosuppressive (60.9 ± 0.01%), curative (85.1 ± 0.04%) and prophylactic (74.6 ± 0.03%) activities in *P. berghei* NK65-infected mice at 1200 mg/kg [119]. Additionally, the activity of three anthraquinone compounds – digitolutein, rubiadin 1-methyl ether, and damnacanthal isolated from the stem bark and the root of the plant against *P. falciparum* have been demonstrated in vitro. The number of parasites significantly decreased in a dose-dependent manner, and 100% inhibition was recorded with 30–40 μg of each compound [122]. In a similar manner, the two triterpenic acids – ursolic and oleanolic acids isolated from the plant showed very good to moderate antiplasmodial activity with IC_50_ values of 3.1 ± 1.3 and 15.2 ± 3.4 µg/ml, respectively [123].

*Persea americana* Mill. is a tropical plant that produces an edible fruit called avocado. The plant is used in ethnomedicine in Nigeria and other parts of Africa for the treatment of different health conditions including monorrhagia, rheumatism, stomach ache, high blood pressure, diarrhoea, bronchitis, diabetes and malaria [23, 80, 81]. The curative and suppressive antimalarial activities of extract and fractions of *P. americana* leaf have been demonstrated in vivo [176]. In the curative model, the extract produced inhibition (*p* < 0.05) of parasitaemia in a dose-dependent manner. The inhibition produced by 400 mg/kg of the extract (methanol in water) was 52.16 ± 2.77%, similar to that of the standard drug artemisinin-combination therapy (ACT) with 69.04 ± 3.02% inhibition. The extract produced significant (*p* < 0.05) chemosuppression (55.00 ± 0.06%) in parasitemia. Similarly, ethyl acetate, hexane, and aqueous fractions of the plant produced significant (*p* < 0.05) chemosuppressive effect by 40.00 ± 0.05, 56.03 ± 0.07 and 64.01 ± 0.08%, respectively at 200–400 mg/kg. However, the effects of the fractions were lower than the standard drug ACT (70.00 ± 0.06%) with only the aqueous fraction (64.01 ± 0.08%) producing a similar activity. The avocadenols, 1, 2, 4-trihydroxyheptadec-16-ene and 1, 2, 4, 15- tetrahydroxyheptadecane-6, 16-diene isolated from *P. americana* seeds showed promising antiplasmodial activity when investigated for their antiplasmodial effect in vitro [177].

*Vernonia amygdalina* Delile is a perennial rainforest herb which is commonly used as a vegetable in preparation of the popular bitter leaf soup as well as for other medicinal purposes in folk medicine in Nigeria [216]. Extracts of the herb have been utilised in ethnomedicine for the treatment of fevers, hiccups, stomach disorders, kidney problems, and malaria [217, 218]. The antimalarial effects of *V. amygdalina* have been reported. Omoregie et al. [137] demonstrated the in vitro and in vivo activities of ethanolic, aqueous and hydroethanolic (50:50) leaf extracts of the plant. In the in vitro study, the ethanolic extract produced the highest (*p* < 0.05) antiplasmodial activity (IC_50_ = 9.83 µg/ml) against *P. falciparum* 3D7 strain. Also, the ethanolic extract was significantly active in vivo against *P. berghei*, dose-dependently, with maximum activity observed at 1,000 mg/kg (82.3% % inhibition). In a 4-day chemosuppression test, a parasitaemia suppression of 67% in *P. berghei*-infected mice was demonstrated following oral administration of methanolic extract of the plant at a dose of 1,000 mg/kg [138]. Also, an aqueous extract of the plant administered orally to *P. berghei*-infected mice at 125 mg/kg reduced the parasitaemia by 63% [139]. Various compounds including steroidal saponins vernoniosides A1, A2, A3, A4 and B1, as well as sesquiterpenes vernodalin, vernodalol, vernolide and hydroxyvernolide isolated from the leaves of the plant have been shown to possess varying degrees of antimalarial effects in vitro. The compounds had antiplasmodial activities with IC_50_ between 4.0 and 46.1 µg/ml and vernodalin was observed to be the most potent (IC_50_ = 4.0 µg/ml) [140].

*Xylopia aethiopica* (Dunal) A. Rich is a sweet-smelling evergreen medicinal plant usually found in the forest-Savannah zone and the rainforest region of Africa [219]. Various parts of the plant including fruit, leaves, stem, stem bark and root are utilised in folk medicine for the treatment of different diseases and health disorders such as rheumatism, asthma, infertility, dysentery, epilepsy, candidiasis, fever, and malaria [220–222]. The in vitro antimalarial effect of the volatile oil of *X. aethiopica* seed was evaluated against *P. falciparum* 3D7 strain, a moderate activity against the malaria parasite was observed with an IC_50_ of 14 µg/ml [124]. In a similar vein, the antimalarial potential of the kaurene diterpene compound xylopic acid isolated from the fruit of the plant was assessed in *P. berghei*-infected mice and it showed promising activity (*p* < 0.05), comparable to that of the standard drug artemether/lumefantrine [125].

In addition to the plants above, the antiplasmodial activity of the underlisted plants have been evaluated in vitro and/or in vivo and are summarized in Table 2: *M. indica*, *C. limon*, *C. citratus*, *L. inermis*, *N. latifolia*, *A. altilis*, *C. longa*, *T. cacao*, *L. alata*, *C. nucifera*, *S. acuta*, *Z. zanthoxyloides*, *C. aurantifolia*, *P. guajava*, *K. ivorensis*, *A. nilotica*, *B. coccineus*, *W. indica*, *A. leiocarpus*, *A. compressus*, *V. paradoxa*, *Z. officinale*, *P. kotschyi*, *A. comosus*, *O. gratissimum*, *P. africana*, *A. altilis*, *C. sinensis*, *F. exasperata*, *P. nigrescens*, *P. thonningii* and *P. guineense*.

### Conservation status of the medicinal plants

The conservation status of the 62 medicinal plant species reported in this study showed that 30 plants were found to be Least Concern (LC), data for 24 were Not Evaluated (NE), 4 each were both Data Deficient (DD) and Vulnerable (VU) i.e., they meet any of the criteria A-E for Vulnerable [57, 223]. Hence, this implies that the VU plants are more likely to go extinct in the wild [223]. Findings from this present study showed that none of the documented indigenous medicinal plant species was found to be Near Threatened (NT), Endangered (EN), Critically Endangered (CR), Extinct in the Wild (EW) and Extinct (EX) suggesting that they have not been overexploited. However, conservation strategies should be intensified to preserve and prevent the plants, especially the VU, from becoming Endangered or Extinct so as to ensure their sustainable availability and biodiversity. This can be achieved by planting these trees through afforestation and forestation programmes to guarantee the sustainable use of the exploited plant taxa [224, 225].

## Conclusion

This is the first comprehensive ethnobotanical study carried out in the region revealing important medicinal plant taxa diversity and ethnomedicinal knowledge held by the TMPs. The current study highlighted the use of indigenous medicinal plants for malaria treatment and fills a gap in our ethnomedicinal knowledge about Kwara State and Nigeria in general. We showed that folk medicine is still being practiced in the State which harbours a wide variety of ethnoflora. A total of 62 medicinal plant species including 13 new plants used for malaria treatment in the State were identified. Although, these medicinal plants are usually combined as recipes for herbal preparations, many of them and their isolated phytocompounds have shown in vitro and/or in vivo antiplasmodial activities against *P. falciparum* and *P. berghei* while some are yet to be evaluated.

Due to their high percentage citation frequency, *M. indica*, *E. chlorantha*, *A. boonei*, *C. citratus* and *N. latifolia* were identified as the most widely used medicinal plants for malaria treatment in folk medicine among the TMPs thus, have great potential to be used in further ethnopharmacological research. This could help contribute to the provision of “*Good Health and Well-being*”, Goal 3 of the United Nations Sustainable Development Goals (UN SDGs), for the hundreds of millions of people infected with the human malaria parasites, and the achievement of the WHO Global Technical Strategy for Malaria goal – “*reduction of global malaria burden by 90% in 2030*”.

Altogether, the data in this current study contributes to both national and international efforts aimed at documenting the local use of indigenous medicinal plants with antimalarial potentials and provides preliminary information for future pharmacological, toxicological and conservation studies.

### Supplementary Information

**Additional file 1.** 

**Additional file 2: Table S1.** Sociodemographic details of informants (*n* = 35).

**Additional file 3: Table S2.** Ethnobotanical data including plant names and voucher numbers of the identified indigenous medicinal plants.

## Data Availability

The data generated or analysed during this study are included in this article.

## Abbreviations

CITES: Convention on International Trade in Endangered Species
CR: Critically Endangered
DD: Data Deficient
EN: Endangered
EW: Extinct in the Wild
EX: Extinct
FHI: Forest Herbarium Ibadan
FRIN: Forestry Research Institute of Nigeria
IC_50_: Half maximal inhibitory concentration
IUCN: International Union for Conservation of Nature
LC: Least Concern
LGA: Local Government Area
LUCRID: Landmark University Centre for Research, Innovation and Discoveries
NE: Not Evaluated
NIPRD: National Institute of Pharmaceutical Research and Development
NT: Near Threatened
TMP: Traditional medicine practitioner
UBH: University of Benin Herbarium
VU: Vulnerable
