# Supplementary material for: Indigenous medicinal plants used in folk medicine for malaria treatment in Kwara State, Nigeria: an ethnobotanical study
Source: BMC Complement Med Ther. 2023 Sep 16;23:324. doi: 10.1186/s12906-023-04131-4 (PMC10504731; doi:10.1186/s12906-023-04131-4)
Supplement: Supplementary file 1 — Additional file 1. [file 12906_2023_4131_MOESM1_ESM.docx]

**Supplementary 1**

Semi-structured questionnaire employed to collect ethnobotanical information from the traditional medicine practitioners (TMPs) including their knowledge of plants used in the treatment of malaria, methods of herbal preparation and mode of administration

**Section A (Socio-demographic Characteristic)**

Age…… Sex/Gender…………... Town/Village………………………………….

LGA………………………………. Senatorial District…………………………….

Religion…………………………… Level of Education…………………………....

**Section B (Questions based on the objective of the study)**

1. Are you a traditional medicine practitioner?

2. How did you become a traditional medicine practitioner?

Is it a family profession? Did you learn it? If yes, how did you learn it?

Were you taught? If yes, how were you taught?

3. How long have you been practicing traditional medicine (Years of experience)?

**Section C**

1. How do you see malaria? What is malaria to you? What can you say about malaria?

2. What are the likely symptoms of malaria?

3. Do you think there are cure for malaria?

4. Are plants/herbs used to treat malaria?

5. What plants do you use for malaria treatment?

6. What part of the plants do you use for malaria treatment?

- Stem bark, roots, leaves, flowers, seeds, whole plant etc.

7. Why do you use particular parts of the plants?

8. How do you use the plants? What are the processes followed in preparing the malaria treatment/cure?

- Do you blend and make a juice out of it/them?

- Do you boil the parts of the plants in water (decoction)?

- Do you boil the parts of the plants in alcohol (decoction)?

- Do you steep (soak) it/them in alcohol? If yes, for how long and why do you choose to soak it/them in alcohol?

- Do you steep (soak) it/them in water? If yes, for how long and why do you choose to soak it/them in water?

9. What other ways can be used in preparing the malaria treatment/cure?

10. Do you use the plants singly or in combination of others?

11. How is the preparation administered? Is there age difference in dosage?

- Is it administered orally?

- Is it by steam inhalation?

- Do they bathe with the decoction water?

12. What other ways can the treatment be administered?

13. Is the treatment/plant preparation gender biased?

14. How will you evaluate this treatment for cure for malaria?

15. Are there any known/reported side effect?
