# Supplementary material for: Indigenous medicinal plants used in folk medicine for malaria treatment in Kwara State, Nigeria: an ethnobotanical study
Source: BMC Complement Med Ther. 2023 Sep 16;23:324. doi: 10.1186/s12906-023-04131-4 (PMC10504731; doi:10.1186/s12906-023-04131-4)
Supplement: Supplementary file 2 — Additional file 2: Table S1. Sociodemographic details of informants (n = 35). [file 12906_2023_4131_MOESM2_ESM.docx]

**Supplementary 2**

**Supplementary table S1** Sociodemographic details of informants (*n* = 35)

| Variables | Categories | Frequency | Percentage (%) |
| --- | --- | --- | --- |
| Gender | Male | 10 | 28.57 |
|  | Female | 25 | 71.43 |
|  |  |  |  |
| Age group | <25 years | 1 | 2.86 |
|  | 25-47 years | 7 | 20.00 |
|  | 48-69 years | 18 | 51.43 |
|  | ≥70 years | 9 | 25.71 |
|  |  |  |  |
| Level of education | None | 17 | 48.6 |
|  | Primary school | 6 | 17.1 |
|  | Secondary school | 7 | 20.0 |
|  | College of Education | 2 | 5.7 |
|  | Polytechnic | 3 | 8.6 |
|  |  |  |  |
| Years of experience | ≤10 | 4 | 11.43 |
|  | 11-25 | 7 | 20.00 |
|  | 26-40 | 13 | 37.14 |
|  | ≥41 | 11 | 31.43 |
|  |  |  |  |
| Religious affiliation | Christianity | 6 | 17 |
|  | Islam | 27 | 77 |
|  | Traditionalism | 2 | 6 |
