# Supplementary material for: Indigenous medicinal plants used in folk medicine for malaria treatment in Kwara State, Nigeria: an ethnobotanical study
Source: BMC Complement Med Ther. 2023 Sep 16;23:324. doi: 10.1186/s12906-023-04131-4 (PMC10504731; doi:10.1186/s12906-023-04131-4)
Supplement: Supplementary file 3 — Additional file 3: Table S2. Ethnobotanical data including plant names and voucher numbers of the identified indigenous medicinal plants. [file 12906_2023_4131_MOESM3_ESM.docx]

**Supplementary 3**

**Supplementary table S2** Ethnobotanical data including plant names and voucher numbers of the identified indigenous medicinal plants

| **S/n** | **Local names** | **Common names** | **Scientific names** | **Voucher number** |
| --- | --- | --- | --- | --- |
| 1. | Mongoro | Common Indian mango | *Mangifera* *indica* Linn. | FHI 109451 |
| 2. | Awopa or Dokita igbo | African yellow wood | *Enantia chlorantha* Oliv. | FHI 109950 |
| 3. | Ahun | Stool wood, Pattern wood | *Alstonia boonei* De Wild | FHI 107254 |
| 4. | Ewe tii | Lemon grass | *Cymbopogon citratus* (DC.) Stapf | FHI 605214 |
| 5. | Egbesi | African peach | *Nauclea latifolia* Sm. | FHI 112779 |
| 6. | Oruwo | Brimstone tree | *Morinda lucida* Benth. | FHI 106992 |
| 7. | Ibepe | Pawpaw | *Carica papaya* Linn. | FHI 109462 |
| 8. | Kasu | Cashew | *Anacardium occidentale* Linn. | FHI 109858 |
| 9. | Aganwo or Oganwo | African mahogany | *Khaya ivorensis* A. Chev. | FHI 56845 |
| 10. | Boonii | Gum Arabic tree | *Acacia nilotica* (L.) Delile | FHI 108425 |
| 11. | Amuje wewe or Kanti-kanti | Crimson thyme | *Byrsocarpus coccineus* Schum. & Thonn. | FHI 112950 |
| 12. | Epoara | Sleeping morning | *Waltheria indica* Linn. | FHI 92465 |
| 13. | Koko | Cocoa | *Theobroma cacao* Linn. | FHI 700314 |
| 14. | Laali (Yoruba), Lǎli (Nupe) | Henna tree | *Lawsonia inermis* Linn. | FHI 702616 |
| 15. | Akerejupon or Ajo | Sphenocentrum | *Sphenocentrum jollyanum* Pierre | FHI 108283 |
| 16. | Osan wewe | Lime | *Citrus aurantifolia* (Christm.) Swingle | FHI 110009 |
| 17. | Ayin | African birch | *Anogeissus leiocarpus* (DC.) Guill. & Perr. | FHI 107122 |
| 18. | Idi | Tropical carpet grass | *Axonopus compressus* (Sw.) P.Beauv. | FHI 109977 |
| 19. | Osan-laimu or Osan agan | Lemon | *Citrus limon* (L.) Osbeck | FHI 110008 |
| 20. | Ponhan | Red iron wood | *Lophira alata* Banks *ex* Gaertn. | FHI 109820 |
| 21. | Dongoyaro | Neem | *Azadirachta indica* A.Juss. | FHI 112927 |
| 22. | Owu or Owu akese (Yoruba), Lulu fùkà (Nupe) | Cotton plant | *Gossypium barbadense* Linn. | FHI 107327 |
| 23. | Karandafi or Poroporo | Red sorghum | *Sorghum bicolor* (L.) Moench | FHI 109659 |
| 24. | Okuuku | Giant rattan | *Ancistrophyllum**secundiflorum* (P.Beauv.) G.Mann & H.Wendl. | FHI 50908 |
| 25. | June 12 | Tree marigold or Mexican sunflower | *Tithonia diversifolia* (Hemsl.) A. Gray | FHI 108055 |
| 26. | Emi | Shea tree | *Vitellaria paradoxa* C.F. Gaertn. | FHI 107924 |
| 27. | Ataile | Ginger | *Zingiber officinale* Roscoe | FHI 107935 |
| 28. | Pandoro | Sausage tree | *Kigelia africana* (Lam.) Benth. | FHI 107654 |
| 29. | Arunje | Dragon’s blood tree | *Harungana madagascariensis* Lam. ex Poir. | FHI 107392 |
| 30. | Gilofa or Gurofa | Guava | *Psidium guajava* Linn. | FHI 110937 |
| 31. | Emi gbegiri or Akodinrin | Dry-zone cedar | *Pseudocedrela kotschyi* (Schweinf.) Harms | FHI 106873 |
| 32. | Ọpẹ oyinbo | Pineapple | *Ananas comosus* (L.) Merr. | FHI 58509 |
| 33. | Tude | Powder puff | *Calliandra haematocephala* Hassk. | FHI 45788 |
| 34. | Efirin | Scent basil | *Ocimum gratissimum* Linn. | FHI 111995 |
| 35. | Ewuro | Bitter leaf | *Vernonia amygdalina* Delile | FHI 112924 |
| 36. | Ayan | African mesquite | *Prosopis africana* (Guill. & Perr.) Taub. | FHI 112370 |
| 37. | Mafowokan omomi or Ahon ekun | Mountain Thistle | *Acanthus montanus* (Nees) T. Anderson | FHI 107529 |
| 38. | Akogun (Yoruba), Kwagũ̀gi (Nupe) | Dutchman’s pipe | *Aristolochia ringens* Vahl | FHI 112929 |
| 39. | Gbere (Nupe) | Breadfruit | *Artocarpus altilis* (Parkinson) Fosberg | FHI 110483 |
| 40. | Orombo | Sweet orange | *Citrus sinensis* (L.) Osbeck | FHI 108811 |
| 41. | Agbon | Coconut palm | *Cocos nucifera* Linn. | FHI 109665 |
| 42. | Ataile pupa | Turmeric | *Curcuma longa* Linn. | FHI 106920 |
| 43. | Iya | African copaiba balsam tree | *Daniellia oliveri* (Rolfe) Hutch. & Dalziel | FHI 36952 |
| 44. | Igbaluwere or Ogurobe | Splinter bean | *Entada africana* Guill. & Perr. | NIPRD/H/6412 |
| 45. | Ipin | Sandpaper | *Ficus exasperata* Vahl | FHI 109550 |
| 46. | Obo | Gutta percha tree | *Ficus platyphylla* Del. Holl | FHI 78251 |
| 47. | Orogbo | Bitter kola | *Garcinia kola* Heckel | FHI 109481 |
| 48. | Lapalapa funfun | Bubble bush | *Jatropha curcas* Linn. | FHI 109020 |
| 49. | Ogbesi | Pheasant-berry | *Margaritaria discoidea* (Baill.) G.L. Webster | FHI 43971 |
| 50. | Ogede agbagba | Banana | *Musa paradisiaca* Linn. | FHI 110122 |
| 51. | Ogbo | African Parquetina | *Parquetina nigrescens* (Afzel.) Bullock | FHI 110044 |
| 52. | Nla | Avocado | *Persea americana* Mill. | FHI 109444 |
| 53. | Abafe | Wild bauhinia | *Piliostigma thonningii* (Schum.) Milne-Redh. | FHI 107815 |
| 54. | Iyere | Climbing black pepper | *Piper guineense* Schum. & Thonn. | FHI 112922 |
| 55. | Sigo | Elephant’s sugarcane | *Cussonia barteri* Hochst. Ex A. Rich. | UBHdt/SN/173 |
| 56. | Jelenubenu (Yoruba), Gayà ebá (Nupe) | Coffee senna or septic weed | *Senna occidentalis* (L.) Link | FHI 109866 |
| 57. | Ajarere | Podocarpa leaf | *Senna podocarpa* **(Guill. & Perr.) Lock** | FHI 109903 |
| 58. | Isekotu (Yoruba), Sàngi yèkó (Nupe) | Common wireweed | *Sida acuta* Burm.f. | FHI 112276 |
| 59. | Opon | Cup of water | *Tetracera potatoria* Afzel. ex G. Don. | FHI 105782 |
| 60. | Aridan/Aidan | Soup perfume | *Tetrapleura tetraptera* (Schum. & Thonn.) Taub. | FHI 110141 |
| 61. | Eeru alamo | Ethiopian or Negro pepper | *Xylopia aethiopica* (Dunal) A. Rich. | FHI 108978 |
| 62. | Orin ata | Senegal prickly-ash | *Zanthoxylum zanthoxyloides* (Lam.) Zepern. & Timler | NIPRD/H/7101 |

FHI – Forest Herbarium Ibadan; NIPRD – National Institute of Pharmaceutical Research and Development; UBH – University of Benin Herbarium
